# Field and Laboratory Performance of False Codling Moth, *Thaumatotibia Leucotreta* (Lepidoptera: Troticidae) on Orange and Selected Vegetables

**DOI:** 10.3390/insects10030063

**Published:** 2019-02-28

**Authors:** Abdullah Mohamed Mkiga, Samira Abuelgasim Mohamed, Hannalene du Plessis, Fathiya Mbarak Khamis, Sunday Ekesi

**Affiliations:** 1 Plant Health Division, International Centre of Insect Physiology and Ecology (ICIPE), Nairobi 00100, Kenya; sfaris@icipe.org (S.A.M.); fkhamis@icipe.org (F.M.K.); sekesi@icipe.org (S.E.); 2 Unit for Environmental Sciences and Management, North-West University, Potchefstroom 2520, South Africa; Hannalene.DuPlessis@nwu.ac.za

**Keywords:** incidence, ripe, mature, fruit, oviposition, preference, offspring, survival

## Abstract

False codling moth (FCM), *Thaumatotibia leucotreta* is a key pest of citrus orange and other plants causing fruit loss through larval feeding. Although this pest is native to sub-Saharan Africa little is known on its performance on orange and vegetables in Kenya and Tanzania. Our objective was to assess the incidence, oviposition preference and offspring performance of FCM on orange and vegetables, namely, okra, African eggplant, chili and sweet peppers. A higher percentage of orange with FCM damage symptoms was recorded from the ground than from the tree sampled fruit. However, FCM larval incidence was higher for the latter (tree sampled fruit). The highest FCM larval incidence amongst the vegetables was recorded on African eggplant (12%) while the lowest was on okra (3%). Orange was the most while African eggplant was the least preferred for oviposition by FCM. Among the vegetables tested, strong oviposition preference was found for sweet pepper; however, larval survival was lowest (62%) on this crop. Highest larval survival (77%) was recorded on orange. Most demographic parameters (i.e., intrinsic rate of increase, doubling time) were comparable among the studied host plants. The results are discussed in line of FCM management.

## 1. Introduction

Fruit and vegetable production are important source of income for East African growers. Production of these crops is, however, constrained by insect pests and diseases resulting in yield loss and poor quality. The false codling moth (FCM), *Thaumatotibia leucotreta* (Meyrick) (Lepidoptera: Tortricidae), one of the pests of these crops, is native to sub-Saharan Africa [1] and has been recorded on 24 cultivated and 50 wild species in different plant families [2]. It is a key pest of citrus (Rutaceae) [3,4], avocado, *Persea americana* (Mill) (Lauraceae) [5] macadamias, *Macadamia* spp. (Proteaceae) [6] and cotton, *Gossypium* spp. (Malvaceae) [1]. *Thaumatotibia leucotreta* is a multivoltine pest [7] which does not enter diapause leading to year-round overlapping generations on host plants [8]. The female moths lay eggs on fruit, often near the stylar end [9]. The hatched larvae penetrate and feed inside the fruit resulting in fruit dropping. Damage symptoms caused by *T. leucotreta* vary with the host plant. For example, scull on avocado [5] and a yellowish-brown rind around a penetration hole on citrus orange [4] have been documented. Larval incidence on orange can be up to 75% [10]. In addition to direct losses, *T. leucotreta* infestations also cause financial losses due to quarantine restrictions imposed on exporting countries and detection of a single larva can result in rejection of an entire consignment [9].

Although South Africa and Egypt are the largest citrus producing countries in Africa, Tanzania and Kenya are considered as the leading countries in citrus production in East Africa [11]. Citrus production in Tanzania is largely concentrated on the North East Coast. The main production areas are in Tanga and Coast region, followed by Morogoro, Mwanza and Ruvuma. In Kenya, citrus production is concentrated in Coast, Eastern and Rift valley provinces [12]. Although *T. leucotreta* has been reported in Kenya and Tanzania [13], little is known about the larval incidence of the pest especially during the citrus orange fruit harvesting period. Orange is produced from low to high altitudes in these countries. Altitudinal gradients and vegetation had been reported to influence distribution and abundance of moths [14,15,16], which are highly diverse and ecologically important herbivorous insects [17]. Odanga et al. [13] reported similar *T. leucotreta* infestations on avocado grown at different altitudinal gradients in Kenya and Tanzania. Knowledge on the effect of altitude on *T. leucotreta* infestation on orange will contribute to management of the pest. The incidence of the pest on other crops which may serve as alternative host crops between successive orange fruiting seasons is not well known.

The ovipositional preference and offspring performance of *T. leucotreta* on orange in a laboratory study was reported by Love et al. [18]. The ovipositional preference of the pest on orange and vegetables has not been determined. According to Thompson and Pellmyr [19], the plant selection made by egg laying females may often provide the initial basis for divergence of insect populations onto different plant species and it may drive the evolution of some plant defences. Developmental biology and adult life parameters of *T. leucotreta* reared on artificial diet have been reported [8,20,21,22] and to a limited extend on orange, grapes and apple [23]. However, no detailed study on the offspring performance of *T. leucotreta* on other key host plants has been reported. The field dynamics of *T. leucotreta* in a mixed cropping system, a common practice in sub-Saharan Africa, need to be investigated to develop better management strategies. The aims of this study were therefore to determine *T. leucotreta* larval incidence on ripe orange as well as on mature vegetables of okra (*Abelmoschus esculentus* (L.) Moench var. Clemson), African eggplant (*Solanum aethiopicum* L., var. Tengeru white), chili pepper (*Capsicum anuum* L., var. Jalapeno) and sweet pepper (*Capsicum anuum* L., var. California Wonder). These vegetables are mainly grown near or within orange orchards. The developmental performance and life table parameters of the pest on three solanaceous vegetables *viz*. chili, sweet pepper and African eggplant were also determined and compared to that of orange, the most common host.

## 2. Materials and Methods

### 2.1. Study Sites

**Figure 1:**
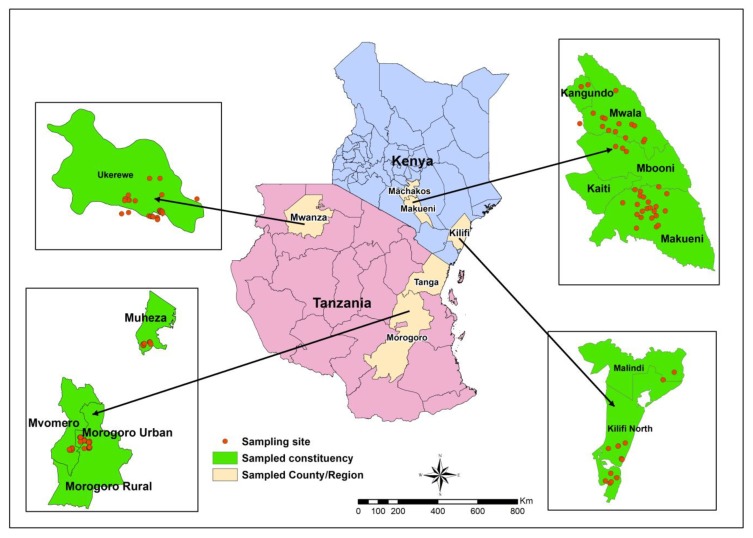
Study sites within Machakos, Makueni and Kilifi in Kenya, Mwanza, Morogoro and Tanga in Tanzania.

Field surveys were carried out between June and September 2017. In Kenya, the survey was conducted in three citrus producing areas namely Kilifi (3°13′6.3″ S, 40°6′54.49″ E), Makueni (1°47′11.38″ S, 37°57′51.99″ E) and Machakos (1°16′1.23″ S, 37°19′12.64″ E) representing low (0–500 masl), mid (501–1200 masl) and high (1201 masl and above) altitudes, respectively (Figure 1). Rainfall in these regions is bimodal. The short rains occur from November to December and the long rains are between March and May. In Tanzania, the study was carried out in Tanga (5°5′19.95″ S, 39°6′8.36″ E), Morogoro (8°3′50.31″ S, 36°57′14.79″ E) and Mwanza (2°52′40.81″ S, 32°43′5.3″ E) regions (Figure 1) representing low, mid and high altitudes respectively. The rainfall pattern in the former and latter region is bimodal and occurs during the same months as that in the Kenyan sites. Morogoro lies in a transition zone between a monomodal and bimodal rainfall pattern.

### 2.2. Assessment of Thaumatotibia leucotreta Incidence on Orange in Kenya and Tanzania

Ripe orange fruit sampling was conducted from June to September 2017 in the three rainfed citrus producing areas of Kenya and Tanzania (Figure 1). The fruit were sampled from 25 orchards in each of the selected altitudes in the two countries. Ten trees were randomly selected from each orchard, from which 10 fruit were sampled for each sampling method. Each sampling method was executed on each of the selected trees. Firstly, fruit were sampled from the ground, followed by sampling from the tree without shaking and lastly by shaking the branches of the tree. In total, 100 fruit were sampled per method per orchard. *Thaumatotibia leucotreta* damage symptoms were recorded for each fruit, washed using a non-caustic liquid dish washing soap and incubated for three weeks. The incubated fruit were dissected and the *T. leucotreta* larvae or pupae counted. The last instar larvae collected were placed in plastic containers (Kenpoly) (2 L) with a thin layer of soft sand covering the bottoms. Smaller larvae were transferred to glass jars containing an artificial diet developed by Moore et al. [24] and reared until pupation. Identification of *T. leucotreta* adults enclosing from the pupae were confirmed using standard keys. The percentage of orange fruit with *T. leucotreta* damage symptoms as well as percentage fruit infested with *T. leucotreta* larvae were calculated.

### 2.3. Assessment of Thaumatotibia leucotreta Incidence in Vegetables from Morogoro, Tanzania

Vegetable sampling was conducted at Mlali ward in Morogoro rural (Figure 1, Table 1) between November and December 2017 following the orange fruiting season. The study site was determined by the availability of vegetable fields near the citrus orchards. Eight fields planted with only one vegetable crop were selected for sampling. These fields were at least 0.1 ha in size and planted with okra, sweet peppers, chili pepper, and African eggplant respectively. The fields were selected on western side and within 100 m from orchard(s). Three hundred matured vegetables per species were randomly collected from each field. The vegetable samples were incubated separately in plastic containers (Kenpoly) (2 L) covered with fine mesh at the top for ventilation. These containers were kept in a laboratory at ambient conditions (23–28 °C) for three weeks before dissection of the vegetables and counting of *T. leucotreta* larvae or pupae. Percentage *T. leucotreta* larval incidence was calculated only. Since *T. leucotreta* damage symptoms on vegetables were not as clear as on oranges, assessment of the percentage of vegetables with damage symptoms caused by this pest, was excluded from the study.

### 2.4. Laboratory Performance of Thaumatotibia leucotreta on Orange and Solanaceous Vegetables

#### 2.4.1. Insect Cultures and Host

Insects were obtained from the mass rearing room at the Animal and Quarantine Containment Unit at the International Centre of Insect Physiology and Ecology (*icipe*), Nairobi, Kenya. This colony was established from field-collected larvae from citrus orange, *Citrus sinensis* L. var Washington Navel fruit in Wote, Makueni county (1°47′11.38″ S, 37°57′51.99″ E) and had been maintained continuously for over 33 generations. The colonies were maintained on artificial diet described by Moore et al. [24] and kept in preserve jars (corked with cotton wool). The procedures for diet preparation and the inoculation of eggs and larvae as well as moth collection were adopted from Opoku-Debrah et al. [25] with slight modifications as described below. The diet in the jars was autoclaved at 121 °C for 20 min then cooled in a laminar-flow cabinet. To obtain eggs, newly emerged moths were transferred to a small rectangular Perspex cage (37 × 22 × 6 cm). The cage was made at *icipe*. A wax paper sheet was inserted into the cage through a slit in the floor to serve as an oviposition substrate for the moths. Wet cotton wool as a source of water for the moths, was plugged into a hole cut at one side of the cage. The oviposition sheet was removed daily, cut into equal pieces, surface-sterilized with 25% formaldehyde (35%–40%, *v*/*v* stock solution, Minema^®^) and placed onto the diet for egg hatching. The neonates fed on the diet and pupated in the cotton wool plug, which was then transferred into a wooden moth emergence box (40 × 40 × 40 cm) made at *icipe*. Eclosing moths were attracted through an exit hole on the side of the emergence box by light, entering through glass vials (32.6 cm^3^). The colony was maintained at 25.0 ± 2.0 °C, 60% relative humidity and 12-h photoperiod (L12:D12).

#### 2.4.2. Oviposition Preference of *Thaumatotibia leucotreta* for Orange and Solanaceous Vegetables

##### Choice Test

Newly emerged naive *T. leucotreta* moths were obtained from a colony reared on artificial diet. The moths were sexed based on the presence of densely packed black fringes of hair on the hind tibia of male *T. leucotreta* as described by Gilligan et al. [1]. One male-female *T. leucotreta* pair was placed in a vial (32.6 cm^3^) closed with moist cotton wool to serve as a source of water [25] and kept for 24 h to mate. A choice test was conducted by placing one fruit each of orange, African eggplant, chili and sweet peppers per corner of the experimental cage (50 × 50 × 50 cm). The cage was also made at *icipe* and had two openings (diameter 10 cm) on two opposite sides. One of the openings was covered with netting cloth; and the other was fitted with a fine netting sleeve for easy handling of the insects and fruit [26]. Eight moths (1:1 ♀:♂) were released at 20:00 h in the cage and left to oviposit for four hours. The fruit were then removed and the number of eggs on each fruit was counted. The fruit were incubated as described above. The experiment was replicated 12 times, for each replicate the position of the fruit was rotated ensuring that every fruit type was placed in each position of the cage an equal number of times.

##### No-choice Test

The no-choice test was done similar to the choice test but with only one type of fruit placed separately per Perspex cage (20 × 15 × 15 cm). Four moths were released in each cage. A replicate consisted of four cages with one fruit per cage. This no-choice test was replicated 12 times.

### 2.5. Developmental Duration and Larval Mass of Thaumatotibia leucotreta Reared on Orange and Solanaceous Vegetables

#### 2.5.1. Insect Colonies and Hosts

Eggs obtained from the rearing colony were incubated in 2 L plastic containers (Kenpoly) until hatching. Two neonate larvae were transferred onto individual fresh fruit of orange, var Washington Navel, African eggplant, chili and sweet peppers of similar cultivars as above, collected from farmers’ fields at Wote, Makueni and Kangundo, Machakos. The inoculated fruit were incubated until final instar larvae exited. The larvae from each fruit type were transferred into separate ventilated plastic containers (Kenpoly) (2 L) with a thin layer of sterilized soft sand for pupation. The containers with the pupae were placed in separate emergence boxes. Adult collection was done following the procedures described above.

#### 2.5.2. Egg Stage

Four Perspex oviposition cages (37 × 22 × 6 cm) were used to obtain *T. leucotreta* eggs from moths of which larvae were reared on the respective host plants, *viz*. orange, African eggplant, chili and sweet peppers. Newly emerged moths were transferred to separate cage containing wax paper sheet for oviposition. Wax paper sheets with eggs were removed and cut into four strips containing at least 100 eggs. The eggs were counted under a stereomicroscope (Leica WILD M3Z). Each strip of wax paper with eggs from moths for each host plant was individually placed in plastic containers (Kenpoly) (2 L) to allow for hatching. The eggs were checked under a microscope twice a day for emergence of neonates. Egg incubation period was recorded as time in days taken for 50% of eggs to hatch as described by Mkiga and Mwatawala [27]. The experiment was replicated four times.

#### 2.5.3. Larval Stage

Duration of *T. leucotreta* larval development was determined on each host fruit type. Upon hatching, a cohort of 100 one day old neonates originating from eggs laid by *T. leucotreta* reared from orange, African eggplant, chili and sweet pepper were inoculated onto a fruit placed in a plastic container following the procedures described by Love et al. [18]. A total of 50 fruit for each replicate were used per each host. The inoculated fruit were incubated individually in ventilated plastic containers (Kenpoly) (2 L) on sterile sand. The fruit were observed daily until final instar larval exited from the fruit. Duration of *T. leucotreta* larval development was recorded as time in days taken for 50% of final instar larvae to exit from the fruit for pupation. The experiment was replicated four times.

To determine the effect of the rearing host on larval mass, recently exited *T. leucotreta* larvae from each host plant were weighed individually using a digital analytical balance (Sartorius CPA225D, Germany). Ten larvae were weighed per replicate for each host plant and the experiment was replicated four times.

#### 2.5.4. Pre-Pupal Stage

To obtain final instar *T. leucotreta* larvae from each host, 300 neonates originating from eggs laid by *T. leucotreta* reared from orange, African eggplant, chili and sweet pepper were transferred onto orange fruit and the respective vegetables. The neonates were collected following the procedures described above. A cohort of 100 final instar larvae exited from each fruit type on the same day were introduced into the four ventilated plastic containers (25 larvae/container) containing a thin layer of soft sterilized sand as pupation media. The larvae were monitored every 12 h until cocoon formation. Duration of the pre-pupal stage was recorded as time in days taken for 50% of final instar larvae to form cocoons. The experiment was replicated four times and each replicate contained 100 larvae.

#### 2.5.5. Pupal Stage

Pupae were obtained by rearing larvae, inoculated as neonates onto the fruit of each host until cocoon formation following the procedures above. A cohort of 100 cocoons were carefully collected and placed in a Perspex cage (15 cm × 15 cm × 10 cm) manufactured at *icipe* and monitored daily for adult eclosion. Pupal duration was recorded as time in days taken for *T. leucotreta* adults to eclose from 50% of the cocoons.

#### 2.5.6. Survival of *Thaumatotibia leucotreta* Reared on Orange and Solanaceous Vegetables

*Thaumatotibia leucotreta* stage specific survival was determined using the cohorts from the developmental duration bioassays above to calculate the percentage of eggs laid on the wax paper strips for each plant species that successfully hatched, percentage of neonates that successfully developed into final instars larvae, percentage of cocoons formed from the final instar larvae and percentage of adults that eclosed from cocoons.

#### 2.5.7. Adult Life History of *Thaumatotibia leucotreta* Reared on Orange and Solanaceous Vegetables

Adult *T. leucotetra* life history was assessed using a cohort of newly emerged moths from larvae reared on orange, sweet pepper, African eggplant and chili pepper. The sex ratio was determined by counting the number of males and females from a sample of 100 moths emerging from pupae from which the larvae were reared on each host plant species. One male-female pair of moths was kept in a vial (32.6 cm^3^) corked with wet cotton wool serving as a source of water. A total of 15 vials were used for each host. The pre-oviposition period was recorded as the number of days from adult eclosion until the first eggs were laid. The pair of moths were carefully removed from the vial and transferred into a new vial using the protocol described by Opoku-Debrah et al. [25]. The number of eggs laid by the female per vial was counted using a magnifying lens. Mortality of the moths was also recorded daily. The experiments were replicated four times.

The net reproductive rate (R0), intrinsic rate of natural increase (rm), mean generation time (T), doubling time (DT) and finite rate of increase (λ) were calculated using the equations from Carey [28].

### 2.6. Data Analysis

The percentage of orange fruit and vegetables (African eggplant, okra, chili and sweet peppers) with *T. leucotreta* damage symptoms as well as fruit containing *T. leucotreta* larvae were arcsine transformed before analysis. Data on percentage of orange fruit with *T. leucotreta* damage symptoms as well as fruit with larvae of the pest were analysed with a three-way ANOVA followed by Tukey’s HSD test to determine statistically significant differences between countries, altitudes and sampling methods. Data on percentage of infested vegetable fruit of the respective host plants were analysed by means of one-way ANOVA followed by Tukey’s HSD test. *Thaumatotibia leucotreta* development time, larval mass and adult life history parameters were analysed by means of one-way ANOVA followed by Tukey’s HSD test. Data on *T. leucotreta* survival in the developmental experiments were analysed using logistic regression models followed by Tukey’s HSD test. Likelihood ratio test on a GLM (family: negative binomial link: log) was used to analyse the number of eggs laid per fruit in the oviposition preference assays as well as the number of eggs laid per day by each female reared from the respective hosts. Pairwise comparison of Least Squares Means (LSMeans; package lsmeans,’ function ‘lsmeans’) of number of eggs laid on choice and no-choice tests and daily oviposition of moths reared on the respective hosts was done with Tukey’s HSD (α = 0.05). All statistical analyses were performed using R-version (3.5.2) statistical software packages (R Development Core Team [29]).

## 3. Results

### 3.1. Percentage of Orange Fruit with Thaumatotibia leucotreta Damage Symptoms and Larvae

Significantly more *T. leucotreta* damaged fruit (*F* = 39.28, df = 1,444, *p* ˂ 0.001) and higher larval incidence were recorded in Kenya than in Tanzania (*F* = 26.43, df = 1,444, *p* ˂ 0.001) (Table 2). The percentage of fruit with damage symptoms also differed significantly between the respective sampling methods (*F* = 277.43, df = 2,444, *p* ˂ 0.001). Significantly more fruit with symptoms were sampled from the ground compared to those sampled from the tree by branch shaking or without shaking (Table 2). The percentage of damaged fruit sampled from the ground did, however, not differ significantly between low, mid and high altitudes in both countries (Table 2). There were, a significantly lower percentage of damaged fruit found on trees without branch shaking at low, compared to mid and high altitudes in Tanzania but not in Kenya. No significant difference in percentage of damaged fruits sampled after the branches of trees were shaken at low, mid and high altitudes existed in Tanzania but a significantly lower percentage of *T. leucotreta* damaged fruit were sampled at low altitude in Kenya (Table 2). Significantly higher percentages of damaged fruit were also sampled from high and mid altitudes compared to low altitudes (*F* = 31.53, df = 2,444, *p* ˂ 0.001). Significantly fewer larvae were found in damaged fruit sampled from the ground at low compared to high altitudes in Kenya. In Tanzania, the percentage of larval infestation per sampling method was similar, regardless of altitude (Table 2). The percentage larval incidence in fruit sampled from the trees as well as from the ground after branch shaking did, however, not differ significantly at the respective altitudes in both countries. Larval incidence was significantly higher in high and mid compared to low altitude (Table 2).

### 3.2. Incidence of Thaumatotibia leucotreta Larvae in Vegetables

The incidence of *T. leucotetra* differed significantly among the vegetables (*F* = 9.812, df = 3, 28, *p* ˂ 0.001). There was no significant difference in percentage *T. leucotetra* larval infested African eggplant, chili and sweet pepper. Significantly fewer okra was, however, infested compared to the other vegetables (Table 3).

### 3.3. Oviposition Preference of Thaumatotibia leucotreta for Orange and Solanaceous Vegetables

#### 3.3.1. Choice Test

**Figure 2:**
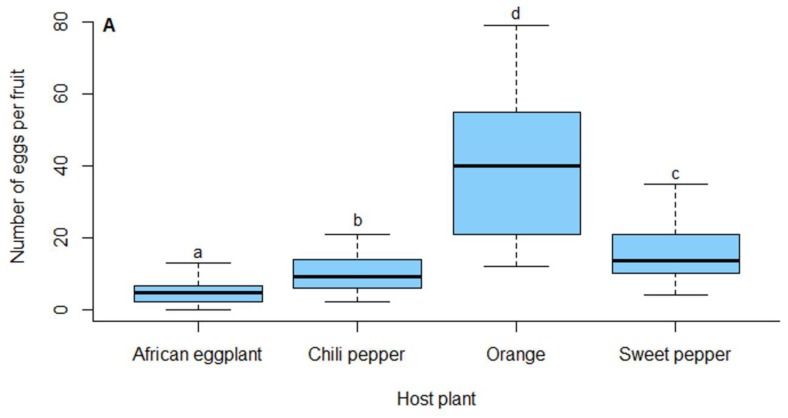

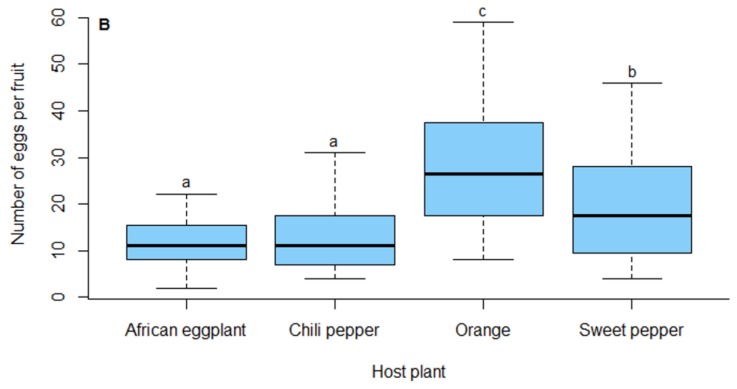
Number of eggs laid by *Thaumatotibia leucotreta* on different host plants in choice (**A**) and no-choice (**B**) experiments. Boxes caped with the same letter are not significantly different (*p* = 0.05, Tukey’s HSD).

The mean number of eggs laid per fruit in the choice test varied significantly among the host plants (LR = 153.21, df = 3, *p* = 0.001) (Figure 2A). The highest number of eggs was recorded on orange followed by sweet and chili pepper. The lowest number of eggs was laid on African eggplant, with no significant difference in oviposition choice between African eggplant and chili pepper.

#### 3.3.2. No-choice Test

The highest number of eggs were also laid on orange in the no-choice test with African eggplant and chili pepper being the least preferred for oviposition (Figure 2B). The number of eggs laid on sweet pepper was significantly less than laid on orange but sweet pepper was significantly more preferred for oviposition than chili pepper and African eggplant (LR = 146.97, df = 3, *p* = 0.001).

### 3.4. Development Time of Thaumatotibia leucotreta Immature Stages and Final Larval Instar Mass when Reared on Orange and Solanaceous Vegetables

**Figure 3:**
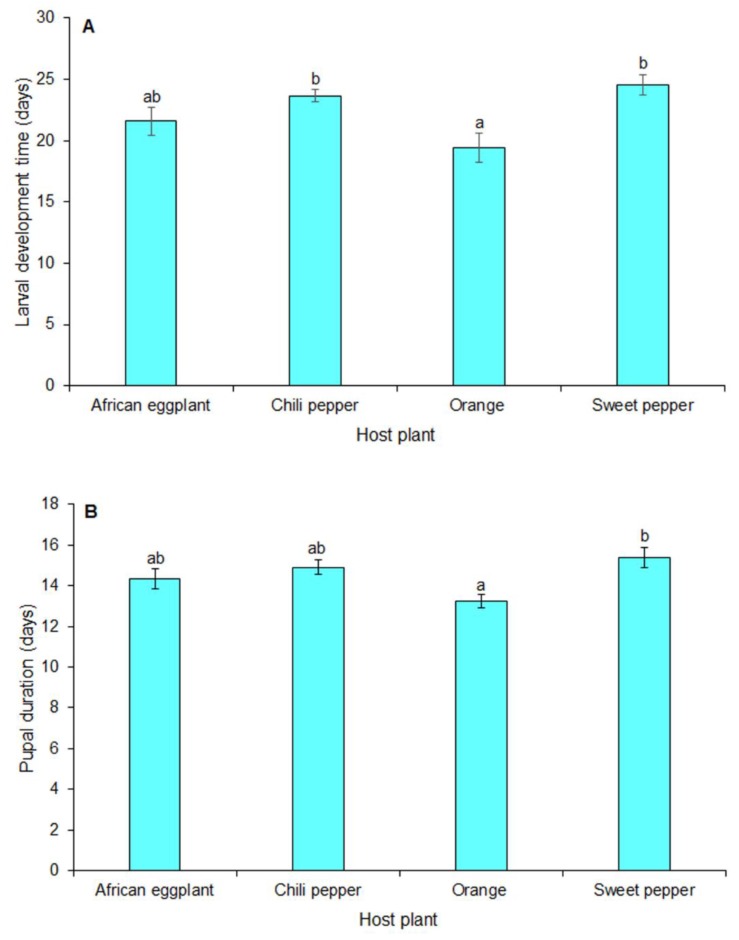
Mean development times (±SE) of *Thaumatotibia leucotreta* larvae (**A**) and pupae (**B**) on orange and solanaceous vegetables. Bars caped by the same letter are not significantly different (*p* = 0.05, Tukey’s HSD).

The host plant on which a specific generation was reared had no significant effect on the egg incubation period of *T. leucotreta* (*F* = 0.13; df = 3, 12; *p* = 0.937). The larval development time (neonates to final instars) differed significantly among the respective solanaceous vegetables (*F* = 5.63; df = 3, 12; *p* < 0.001) (Figure 3A). Development was significantly shorter on orange compared to sweet and chili peppers. Larval development time on African eggplant, did, however, not differ significantly from development time on orange. The prepupal period from larvae reared on the respective host plant species did not differ significantly (*F* = 0.96; df = 3, 12; *p* = 0.444) but duration of the pupae originated from larvae reared on these solanaceous vegetables differed significantly (*F* = 4.84; df = 3, 12; *p* < 0.040) (Figure 3B). Pupal duration from which the larvae were reared on orange was significantly shorter compared to sweet pepper (Figure 3B). There was, however, no significant difference in duration of pupae originating from larvae reared on African eggplant, chili and sweet pepper.

**Figure 4:**
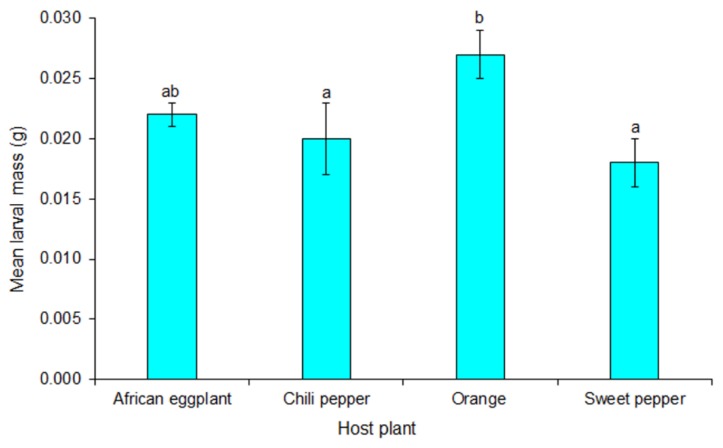
Mean mass (±SE) of final instar *Thaumatotibia leucotreta* larvae from orange and solanaceous vegetables. Bars caped with the same letter are not significantly different (*p* = 0.05, Tukey’s HSD).

Mass of final instar larvae reared on orange was significantly higher compared to larvae reared on vegetables (African eggplant, chili and sweet pepper) (*F* = 5.84; df = 3, 12; *p* = 0.011). It did, however not differ significantly between the vegetables (Figure 4).

### 3.5. Development and Survival of Thaumatotibia leucotreta Immature Stages Reared on Orange and Solanaceous Vegetables

**Figure 5:**
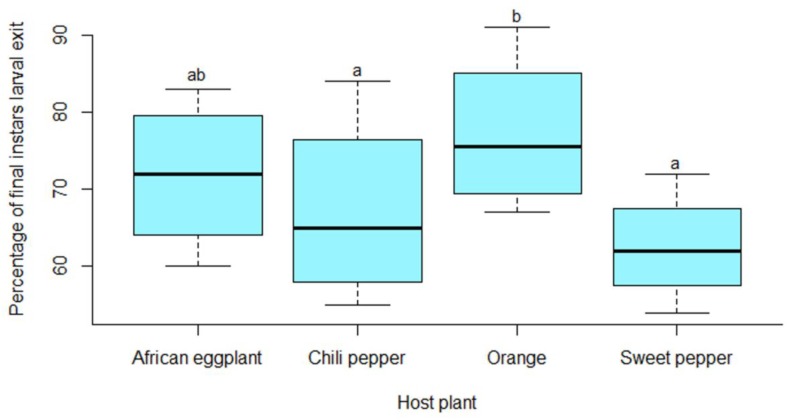
Percentage of surviving *Thaumatotibia leucotreta* larvae reared on orange and solanaceous vegetables. Boxes caped by the same letter are not significantly different (*p* = 0.05, Tukey’s HSD).

The percentage fertility of eggs laid by moths of which larvae were reared on orange, sweet pepper, chili pepper and African eggplant in the previous generation did not differ significantly (χ^2^ = 26.436, df = 3, 12; *p* < 0.916). The percentage of larvae that survived differed significantly between the rearing hosts (χ^2^ = 65.049, df =3,12; *p* ˂ 0.001). A significantly higher percentage of larvae reared on orange survived compared to sweet and chili peppers (Figure 5). The percentage surviving larvae reared on orange and African eggplant did, however, not differ significantly. The percentage pupae that survived from larvae reared on these four host plants did not differ significantly (χ^2^ = 8.018, df = 3, 12; *p* = 0.904) as well as the percentage of adults that eclosed from these pupae (χ^2^ = 16.456, df = 3, 12; *p* = 0.937).

### 3.6. Reproductive Parameters for Thaumatotibia leucotreta Reared on Orange and Solanaceous Vegetables

The host plant on which the larvae were reared did not significantly affect the pre-oviposition period (*F* = 0.08; df =3, 12; *p* = 0.971), oviposition period (*F* = 0.48; df = 3,12; *p* = 0.703), female longevity (*F* = 0.16; df = 3,12; *p* = 0.923) and male longevity (*F* = 0.04; df = 3,12; *p* = 0.990) (Table 4). The fecundity of the moths from larvae reared on the different host plants was, however, significantly different (*F* = 32.32; df = 3,12; *p* ˂ 0.001). Moths originating from larvae that were reared on orange laid significantly more eggs than moths from larvae reared on African eggplant as well as on sweet and chili peppers. Moths from African eggplant reared larvae were also more fecund than those reared on sweet peppers but fecundity of moths reared on African eggplant and chili pepper was similar.

#### 3.6.1. Fecundity per Female per Day

**Figure 6:**
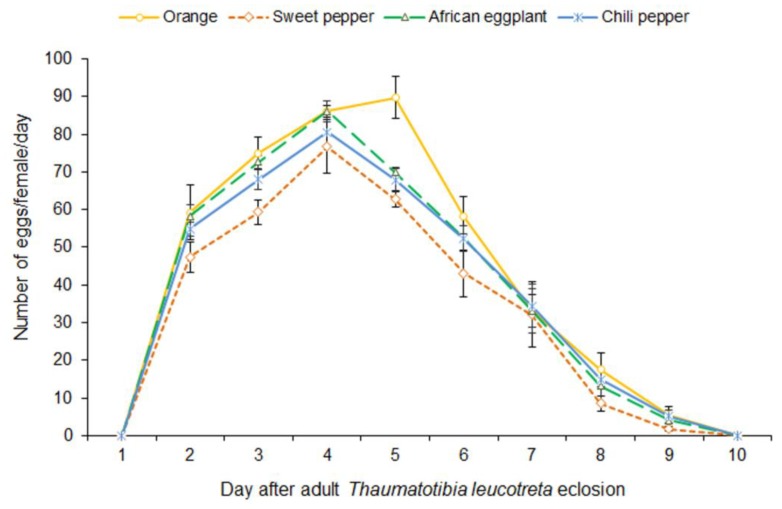
Mean (±SE) daily oviposition of *Thaumatotibia leucotreta* reared from orange and solanaceous host fruit.

There was no significant interaction (LR = 200.0, df = 3, 24; *p* = 0.205) between moth age and host plant in terms of daily oviposition (Figure 6). Host plant used for rearing the larvae did, however, had a significant effect on egg laying (LR = 3556.4, df = 3, *p* ˂ 0.001). Moths from larvae reared on orange laid the most eggs per day. The number of eggs laid per day varied considerably with female age (LR = 229.4, df = 8, *p* ˂ 0.001). The most eggs were laid by moths from larvae reared on orange, five days after moth eclosion. Egg laying for females reared on the other host plants peaked on the fourth day after moth eclosion. On the days where oviposition was high, females from the larvae reared in orange laid more egg followed by females from larvae reared from African eggplant, chili and sweet peppers.

#### 3.6.2. Pairwise Comparison on Fecundity Rate of *Thaumatotibia leucotreta* Reared from Orange and Solanaceous Hosts

Pairwise comparison of the three days where oviposition was highest (Table 5), for *T. leucotreta* reared from orange and solanaceous hosts shows that oviposition of females reared from the tested hosts on day three was comparable. On day four, the females reared from orange oviposited significantly more eggs when compared to each solanaceous host. However, the number of eggs oviposited by the females reared from solanaceous hosts did not differ significantly when compared to each other. On day five, the number of eggs laid by *T. leucotreta* females reared from orange was significantly higher than from females reared on sweet pepper.

### 3.7. Life History Parameters of Thaumatotibia leucotreta Reared from Orange and Solanaceous Vegetables

Net reproductive rate of *T. leucotreta* reared on the respective hosts differed significantly (*F* = 30.61; df = 3, 12; *p* ˂ 0.001) (Table 6). The highest net reproductive rate was recorded for females reared from larvae reared on orange followed by those reared on eggplant while the lowest was from larvae on sweet pepper. Other demographic parameters; mean generation time (*F* = 0.50; df = 3, 12; *p* = 0.688), intrinsic rate of increase (*F* = 1.23; df = 3, 12; *p* = 0.341), doubling time (*F* = 1.43; df = 3, 12; *p* = 0.284) and finite rate of increase (*F* = 1.77; df = 3, 12; *p* = 0.207) were similar for females reared from larvae on the respective host plants.

## 4. Discussion

*Thaumatotibia leucotreta* occurred in all surveyed sites of Kenya and Tanzania from low to high altitudes. The higher percentage of damaged fruit recorded in Kenya compared to Tanzania could be attributed to the different farming practices within each country. In Kenya, most citrus trees are intercropped with peppers and maize which are also recorded as crop hosts of *T. leucotreta* [1,6] while such practices are not common in Tanzania. Pests and diseases are generally controlled by means of pesticide application, which may result in a reduction of the natural enemy populations in the citrus orchards in Tanzania. According to Hofmeyr and Pringle [30], excessive use of broad spectrum chemical pesticides causes pest outbreaks due to disruption of natural enemies and it could also lead to the development of insecticide resistance. The fruit sampled from the ground had a higher percentage of *T. leucotreta* damage symptoms than those sampled from trees (with or without branch shaking). Higher *T. leucotreta* larval incidence was, however, recorded from fruit sampled after shaking of the tree branches. This could be ascribed to *T. leucotreta* laying eggs mainly on fruit while they are still on the trees [31] and the larvae completing their development while the fruit is still on the tree [9]. When the fruit drop to the ground, the larvae would have already completed their development and exited the fruit to pupate in the soil. Lower larval incidence was therefore recorded in the fruit sampled on the ground. Although. *T. leucotreta* infested fruit do often not show damage symptoms, final instar larvae may be present in the fruit mainly in the centre cores. This may have implications for *T. leucotreta* management especially in terms of orchard sanitation and post-harvest treatment. Proper orange orchard sanitation and harvesting should therefore also take in consideration infested fruit that is still on the tree by shaking of the branches. Higher *T. leucotreta* larval incidence on orange fruit was recorded in high than low altitudes of both countries. In contrast, Odanga et al. [13] reported a similar population density and number of infested avocado fruit in different altitudes of Mount Kilimanjaro in Tanzania and Taita Hills in Kenya. This difference could possibly be ascribed to the difference in crop and altitudinal ranges.

Amongst the vegetables sampled, the highest percentage of *T. leucotreta* infested fruit was on African eggplant compared to okra, sweet and chili peppers. This could be due to the lower moisture content of the fruit of this host plant, since infested African eggplant produced very little fluid under field and laboratory conditions. According to Jaenike [32], the suitability of a plant for larval development is a function of many variables, including its chemical and physical properties, microhabitat and degree of infestation. During dissection of fruit, final instar larvae were also mainly found in the central core of orange and placenta of peppers where very little fluid was produced due to insect feeding and decay. African eggplant, okra, sweet and chili pepper serve as reservoirs for *T. leucotreta* when grown in the vicinity or in mixed cropping with orange orchards. These vegetables are also grown throughout the year in irrigated fields which provides a continuous availability of host plants for this pest.

Although vegetables can serve as host plants for *T. leucotreta*, oranges were the most preferred host plant for oviposition in both choice and no-choice tests. The *T. leucotreta* moths do prefer certain orange varieties above others for oviposition. For example, Love et al. [18] reported that Fischer Navels were the least preferred early maturing variety for oviposition. Navel oranges were reported to be more preferred for oviposition than Valencia oranges and guava [33]. Most of the available literature report on the oviposition preference of *T. leucotreta* for different citrus varieties and guava under laboratory and field conditions [18,33]. This study provides additional information on *T. leucotreta* ovipositional preference for and performance on solanaceous vegetables, with chili and sweet pepper more preferred for oviposition than African eggplant.

In general, insects are reported to oviposit on hosts that maximize the performance of their progeny, a hypothesis referred to as “mother knows best” [19,32,34,35]. In this study, oviposition preference by *T. leucotreta* did not mirror host suitability for development of the immatures and survival on these host plants. The sweet and chili peppers which were more preferred than African eggplant for oviposition were less suitable in terms of developmental time, larval mass and survival of immature stages (larvae and pupae) as well as female fecundity and net reproductive rate. The mismatch between preferred vegetable hosts for oviposition and host suitability of peppers could be explored for use as trap crop. A similar finding was reported for the butterfly, *Anthocharis cardamines* (L) (Lepidoptera: Pieridae). The oviposition preference of females did not correlate with the fitness parameter of the offspring reared on 51 populations of two ploidy types of the perennial herb *Cardamine pratensis* L. (Brassicales: Brassicaceae) [36]. The suitability of the African eggplant for development of the immature stages of *T. leucotreta* could be explained by the higher protein and lower moisture content of African eggplant compared to sweet and chili pepper [37,38,39]. Rearing host quality has also been reported to influence the developmental duration of other lepidopteran species. For example, Traore et al. [40] reported the larvae of *Maruca vitrata* (Fabricius) (Lepidoptera: Pyralidae) reared on cowpea flowers to have a short developmental time compared to those reared on the other plant parts. The effect of host plants on the life table parameters is dependent on the quality of certain components, such as carbon, nitrogen and defensive metabolites [41].

In this study, adult life table parameters for moths from larvae reared on the respective host plants are similar except for fecundity and net reproductive rate. Fecundity of *T. leucotreta* ranged from a total of 341 to 415 eggs per female for those reared on sweet pepper and orange, respectively. Both these values are within the range reported for fecundity of *T. leucotreta* from larvae reared on artificial diet [8,25]. Females reared on solanaceous vegetables laid fewer eggs than moths from larvae reared on orange. Daily egg laying peaked at day three and four after eclosion, for females reared on solanaceous vegetables and orange, respectively. *Thaumatotibia leucotreta* oviposition from larvae reared on artificial diet reached a peak, 2 and 3 days earlier than for the females reared on solanaceous vegetables and orange, respectively [8]. Generally, the highest daily fecundity was recorded for females reared from orange while the lowest was for females reared from sweet pepper. Oviposition peaked one day earlier (day three) for those of which larvae were reared on solanaceous vegetables than those reared on orange (day four) after moths emerged.

The intrinsic rate of natural increase, mean generation time, doubling time and finite rate of increase are important biotic parameters of insect performance [42,43]. Considering these parameters, survival of *T. leucotreta* is equal on orange, sweet and chili pepper as well as African eggplant. This has implications for management strategies of the pest. According to Birch [44] the intrinsic rate of increase is a basic parameter for insect population development, which is an indicator of the species developmental speed, longevity and fecundity [28].

Although oranges sampled from the ground had more *T. leucotreta* damage symptoms compared to fruit sampled from the trees, the latter hosted a higher number of *T. leucotreta* larvae. It is therefore recommended that citrus trees should be subjected to gentle shaking prior to fruit harvesting to reduce citrus orange post-harvest fruit decay and minimize indirect losses due to quarantine measures. Infested orange fruit from both ground and tree after branch shaking should be collected and removed using sustainable sanitation measures such as the use of augumentorio which sequesters the moth while conserving any parasitoids. This control tactic should be integrated with other *T. leucotreta* control measures such as the use of biopesticides *viz*. *Cryptophlebia leucotreta* granulovirus (CrleGV), entomopathogenic nematode, *Heterorhabditis bacteriophora*, entomopathogenic fungi, *Metarhizium anisopliae*, *Beauveria bassiana*, attract and kill through the use of Lastcall^®^ and the Sterile Insect Technique. The potential of solanaceous vegetables to act as *T. leucotreta* reservoirs between successive citrus orange seasons for rainfed orange production, the common cropping systems in Kenya and Tanzania were determined. The strong preference of *T. leucotreta* to chili and sweet peppers for oviposition and relatively higher mortality of the larvae on these crops could be explored for management of this pest using attract and kill approaches. Although there is currently a very potent male moth attractant commercially available, a need to identify semio-chemicals that can attract female *T. leucotreta* moths for both monitoring and suppression of the pest, is needed.

## 5. Conclusions

More ripe orange fruit with *T. leucotreta* damage symptoms are on the ground but the larvae are abundant in the fruit still hanging on the tree. Orange is strongly preferred for oviposition and highly suitable for *T. leucotreta* larval survival when compared to solanaceous vegetables. *Thaumatotibia leucotreta* has a strong oviposition preference to sweet and chili pepper compared to African eggplant. African eggplant is, however, highly suitable for larval survival. Fecundity is the only affected adult *T. leucotreta* fitness trait from larvae reared from orange and solanaceous vegetables. The findings from this study will contribute to sustainable management of this pest.

## Figures and Tables

**Table 1:** Vegetable samples collected near citrus orchards for *Thaumatotibia leucotreta* incidence in mid altitude, Mlali, Morogoro, Tanzania.

Village/Orchard	Latitude	Longitude	Vegetables Sampled Nearby the Orchard	Distance from the Orchard
Mlali	S06°56′55.3″	E037°31′55.4″	Sweet pepper, African eggplant, okra, chili pepper	67–72 m
Mlali	S06°56′33.6″	E037°31′59.1″	Sweet pepper, African eggplant, okra, chili pepper	69–75 m
Mlali	S06°57′20.5″	E037°32′02.9″	Sweet pepper, African eggplant, okra, chili pepper	58–69 m
Mlali	S06°57′46.8″	E037°32′14.1’′	Sweet pepper, African eggplant, okra, chili pepper	65–70 m
Mlali	S06°57′35.2″	E037°31′51.8″	Sweet pepper, African eggplant, okra, chili pepper	68–74 m
Mkuyuni	S06°58′41.9″	E037°31′58.0″	Sweet pepper, African eggplant, okra, chili pepper	63–69 m
Kipera	S06°56′14.3″	E037°31′40.2″	Sweet pepper, African eggplant, okra, chili pepper	66–74 m
Vitonga	S06°57′15.9″	E037°29′35.9″	Sweet pepper, African eggplant, okra, chili pepper	55–67 m
Vitonga	S06°58′36.8″	E037°29′23.0″	Sweet pepper, African eggplant, okra, chili pepper	66–72 m
Vitonga	S06°57′52.4″	E037°30′38.5″	Sweet pepper, African eggplant, okra, chili pepper	56–63 m

**Table 2:** Percentage of fruit with *Thaumatotibia leucotreta* damage symptoms (A) and larvae (B) sampled from the ground and from trees (with and without branch shaking) at the respective altitudes in Kenya and Tanzania.

Parameter	Altitude	Fruit Sampling Method	Country		Average
			Kenya	Tanzania	
**A% fruit with damage symptoms**	>1201 masl	Ground	42.16 ± 3.40 h	33.92 ± 3.05 fgh	38.04 ± 2.29 e
		Tree without shaking	12.72 ± 1.69 bcd	9.36 ± 1.35 bc	11.04 ± 1.10 b
		Tree shaking	28.48 ± 2.88 fg	22.08 ± 2.12 def	25.28 ± 1.75 c
	501–1200 masl	Ground	40.24 ± 2.28 gh	28.58 ± 2.72 fgh	34.38 ± 1.91 de
		Tree without shaking	7.72 ± 1.26 abc	5.52 ± 1.96 bc	6.62 ± 0.80 a
		Tree branch shaking	27.56 ± 2.20 efg	21.40 ± 1.95 def	24.48 ± 1.49 c
	0–500 masl	Ground	35.88 ± 1.87 gh	21.76 ± 2.49 def	28.82 ± 1.82 cd
		Tree without shaking	5.76 ± 1.21 ab	2.88 ± 0.76 a	4.32 ± 0.74 a
		Tree shaking	16.08 ± 1.82 cde	14.16 ± 1.69 cd	15.12 ± 1.21 b
Average			24.07A	17.73B	
B% Larval infested fruit					
	>1201 masl	Ground	33.26 ± 3.59 efg	21.00 ± 2.75 cde	27.12 ± 2.41de
		Tree without shaking	16.00 ± 0.99 bcd	12.08 ± 0.75 abc	14.04 ± 0.68 bc
		Tree shaking	46.48 ± 4.38 g	41.80 ± 3.69 fg	44.14 ± 2.85 g
	501–1200 masl	Ground	29.00 ± 3.02 def	16.40 ± 1.84 bcd	22.62 ± 1.96 cd
		Tree without shaking	14.93 ± 1.27 bc	8.96 ± 0.63 abc	11.90 ± 0.82 ab
		Tree shaking	45.04 ± 3.75 g	37.44 ± 3.75 fg	41.24 ± 2.64 fg
	0–500 masl	Ground	15.46 ± 1.80 bc	11.44 ± 1.27 abc	13.40 ± 1.13 b
		Tree without shaking	8.27 ± 0.83 ab	4.92 ± 0.49 a	6.64 ± 0.54 a
		Tree shaking	34.24 ± 3.99 efg	33.00 ± 3.78 efg	33.62 ±2.72 ef
Average			26.93A	20.79B	

Means for *Thaumatotibia leucotetra* damage symptoms and larval incidence in the same column followed by the same lower-case letter and means within a row followed by the same upper case letter do not differ significantly at *p* = 0.05 (Tukey’s HSD).

**Table 3:** Mean percentage of *Thaumatotibia leucotreta* larval infested vegetables sampled from fields near orange orchards in the mid-altitude growing region of Tanzania.

Host Plant	Scientific Name	Percentage Infestation
Okra	*Abelmoschus esculentus*	3.08 ± 0.90 a
Sweet pepper	*Capsicum* spp.	8.72 ± 1.81 b
African eggplant	*Solanum aethiopicum*	12.00 ± 1.46 b
Chili pepper	*Capsicum* spp.	10.96 ± 1.28 b

Means within the column followed by the same letter do not differ significantly at *p* = 0.05 (Tukey’s HSD).

**Table 4:** Sex ratio, oviposition, female longevity, male longevity and fecundity of *Thaumatotibia leucotreta reared on* orange and solanaceous vegetables.

Parameter	Host Plant			
	Orange	Sweet Pepper	African Eggplant	Chili Pepper
* Sex ratio (male: female)	0.38:0.62	0.32:0.68	0.37:0.63	0.34:0.66
* Pre-oviposition period	1.20 ± 0.08	1.21 ± 0.09	1.17 ± 0.12	1.23 ± 0.06
* Oviposition period	8.00 ± 0.4	7.75 ± 0.29	8.00 ± 0.41	7.75 ± 0.25
* Female longevity (days)	16.02 ± 0.89	16.21 ± 0.71	16.61 ± 0.73	15.90 ± 0.82
* Male longevity (days)	15.67 ± 0.69	15.34 ± 0.60	15.51 ± 0.90	15.61 ± 0.81
Fecundity	415.00 ± 3.70c	341.00 ± 5.37a	378.00 ± 8.03b	364.00 ± 3.53b

Means followed by the same letter within the row for fecundity are not significantly different (*p* = 0.05, Tukey’s HSD), * No significant difference between hosts.

**Table 5:** Pairwise comparison on mean number of eggs laid by *Thaumatotibia leucotreta* reared from orange and solanaceous fruit on three peak days of egg laying.

	Orange	Chili Pepper	African Eggplant	Sweet Pepper
Day 3				
Orange	-	0.823	1.000	0.460
Chili pepper		-	0.829	0.928
African eggplant			-	0.459
Day 4				
Orange	-	**0.003**	**0.009**	**0.001**
Chili pepper		-	0.988	0.822
African eggplant			-	0.630
Day 5				
Orange	-	0.6815	0.177	**0.010**
Chili pepper		-	1.000	0.182
African eggplant			-	0.177

*p*-Values indicating significant differences are in bold (Tukey HSD, α = 0.05).

**Table 6:** Life history parameters of *Thaumatotibia leucotreta* recovered from orange and solanaceous vegetables.

Parameter	Host Plant			
	Orange	Sweet Pepper	African Eggplant	Chili Pepper
Net reproductive rate	419.7 ± 3.844c	348.00 ± 5.484a	385.90 ± 7.722b	371.5 ± 3.611b
* Mean generation time	48.19 ± 1.103	48.90 ± 1.301	47.29 ± 0.807	48.42 ± 0.261
* Intrinsic rate of increase	0.13 ± 0.002	0.12 ± 0.003	0.13 ± 0.002	0.12 ± 0.001
* Doubling time	5.53 ± 0.128	5.79 ± 0.162	5.50 ± 0.086	5.67 ± 0.024
* Finite rate of increase	1.40 ± 0.002	1.13 ± 0.004	1.13 ± 0.002	1.12 ± 0.002

Means followed by the same letter within the row indicating the net reproductive rate are not significantly different (*p* = 0.05, Tukey’s HSD), * No significant difference between hosts.
